# Psychometric Properties of the Grief Cognitions Questionnaire for Children (GCQ-C)

**DOI:** 10.1007/s10942-016-0236-0

**Published:** 2016-04-28

**Authors:** Mariken Spuij, Peter Prinzie, Paul A. Boelen

**Affiliations:** 1 Department of Child and Adolescent Studies, Utrecht University, PO Box 80140, 3508 TC Utrecht, The Netherlands; 2 Department of Pedagogical Sciences, Erasmus University Rotterdam, Rotterdam, The Netherlands; 3 Department of Clinical Psychology, Utrecht University, Utrecht, The Netherlands; 4 Arq Psychotrauma Expert Group, Diemen, The Netherlands

**Keywords:** Prolonged-grief-disorder, Grief cognitions, Children, Adolescents, Assessment

## Abstract

Negative thinking is seen as an important mediating factor in the development of prolonged grief disorder (PGD), a syndrome encompassing debilitating symptoms of grief. No measure of specific grief related cognitions is available yet. Based on an adult measure of negative thinking in adults we developed a questionnaire for children, the Grief Cognitions Questionnaire for Children (GCQ-C). This study investigated several psychometric properties of the GCQ-C. Both reliability and validity were investigated in this study, in which hundred fifty-one children and adolescents (aged 8–18 years) participated. Findings showed that items of the GCQ-C represented one underlying dimension. Furthermore, the internal consistency and temporal stability were found to be adequate. Third, the findings supported the concurrent validity (e.g., significant positive correlations with self-report indices of PGD, depression and posttraumatic stress disorder), convergent and divergent validity of the GCQ-C. This study provides further evidence for the importance of negative thinking in PGD in children and adolescents.

## Introduction

There is evidence that, in a significant minority of bereaved children, adolescents and adults, acute grief reactions turn into chronic debilitating distress, blocking reestablishment of normal routines (e.g., Dowdney 2008; Melhem et al. 2007, 2011). This evidence has led to the inclusion of Persistent Complex Bereavement Disorder in the Appendix of the Diagnostic and Statistical Manual of mental disorders [DSM-5; American Psychiatric Association (APA) 2013; for discussions see Boelen and Prigerson 2012; Kaplow et al. 2012; Wakefield 2012]. Consistent with many recent studies (see Kaplow et al. 2012), we refer to this syndrome as prolonged grief disorder (PGD). PGD includes symptoms of separation distress, numbness, avoidance and detachment present to the point of functional impairment at least 6 months following the death of a loved one (Prigerson et al. 2009). To date most research on PGD has been done in adults, but there is a growing body of research done in children and adolescents. There is strong evidence that symptoms of PGD are distinct from symptoms of depression, posttraumatic stress disorder (PTSD) and other anxiety disorders (Boelen and Prigerson 2012; Lichtenthal et al. 2004; Prigerson et al. 2009; Shear et al. 2011; Spuij et al. 2012a, b).

In order to understand why some children cope with loss more easily than others, it is important to identify variables that mediate the development and maintenance of PGD. Given the lack of effective interventions for bereaved youth (Currier et al. 2007), this knowledge can be very helpful for the development of interventions for bereaved children who fail to recover from loss. Recent cognitive behavioral conceptualizations of PGD propose that negative thinking and avoidance behaviors play a significant role in the development and maintenance of the disorder (Boelen et al. 2006; Maccallum and Bryant 2013). Empirical findings, mostly from adults, have confirmed that some bereaved individuals develop negative thinking that contributes to the development of PGD (Boelen and Lensvelt-Mulders 2005; Boelen et al. 2003). However, little is known about negative thoughts associated with emotional distress in bereaved children. Findings of a preliminary study with 30 adolescent girls, aged 13–18 years (Boelen and Spuij 2008), showed that different types of cognitions were significantly associated with symptom levels of PGD and depression; global negative thinking about life and self and catastrophic misinterpretations were most strongly linked with these symptoms. To our knowledge, no research is available about specific grief related negative thoughts in bereaved children under the age of 13. This is remarkable, as many children are confronted with loss (Harrinson and Harrington 2001). Understanding the cognitive profile that is associated with specific grief related psychopathology in children is important for the conceptualization and treatment of this psychopathology.

Despite the widespread idea that alleviation of mood and anxiety symptom requires changes in underlying maladaptive thinking patterns (cf. Beck 1967; Ellis and Grieger 1977; Meiser-Stedman et al. 2009; Treadwell and Kendall 1996), there is a scarcity of measures to assess negative thinking in children who suffer from depression, PTSD or anxiety. Moreover, there is no measure of negative thoughts involved in the development and maintenance of PGD. Examples of measures that do assess negative thinking in youth are the Automatic Thoughts Questionnaire (ATQ, Hollon and Kendall 1980; Kazdin 1990), and the Negative Affectivity Self Statement Questionnaire (NASSQ; Ronan et al. 1994). Campbell et al. (2000) stated that the majority of childhood cognitive measures, including the ATQ, are limited by the fact that they are downward extensions of questionnaires developed for adults. Other measures, like the NASSQ, have different versions for children and adolescents, which is a limitation for e.g. research purposes. In light of these limitations, Schniering and Rapee (2002) developed the Children’s Automatic Thoughts Scale (CATS), a measure specifically designed to assess negative thinking in children and adolescents. The authors first started with a pool of items that was generated from in-depth interviews with children and adolescents with various forms of emotional problems. Items relating to affect or behavior were removed, so only items that reflect cognition remained. The final measure consists of 40 items covering a broad range of negative automatic thoughts (e.g., “There is something very wrong with me”, “I look like an idiot”, “I’m worthless” and “Most people are against me”).

Given the lack of measures for negative thinking in children in general and more specific for bereaved children, our aim was to develop a measure for the latter group. Specifically, the current study sought to enhance knowledge on the role of negative thinking in PGD and other forms of emotional distress among children confronted with the death of a loved one. To this end, as described in more detail below, we constructed the so called Grief Cognitions Questionnaire for Children (GCQ-C) for bereaved children aged 8–18 years, drawing from earlier work on negative cognitions in adult PGD (Boelen and Lensvelt-Mulders 2005). Because of the limitations mentioned by Campbell et al. (2000) about the usefulness of adult measures in children, we asked child psychologists, children and adolescents to help us with the development of this specific child version. We describe this process below. Furthermore, our aim was to evaluate several psychometric properties of this GCQ-C. Specifically, we studied the dimensionality, internal consistency, and temporal stability of the GCQ-C. In addition, we studied several predictions concerning the validity of the GCQ-C. With respect to the concurrent validity, we expected that stronger endorsement of negative cognitions tapped by the GCQ-C would be associated with increased symptom levels of PGD, depression, and PTSD as well as Internalizing and Externalizing Problems. In addition, we expected elevated negative thinking—tapped by the GCQ-C—to be associated with reduced *coping efficacy*, defined as the participant’s own perceived capability to adjust to the loss (cf. Benight and Bandura 2004). With respect to convergent and divergent validity, we expected scores on the GCQ-C to be more strongly associated with cognitions associated with depressive and anxious states (tapped by the subscales social threat, physical threat and personal failure of the CATS; cf. Schniering and Rapee 2002), than cognitions associated with anger (tapped by the subscale hostile intent of the CATS; cf. Schniering and Lyneham 2007). In addition, it was predicted that, compared to the summed scores on the CATS (tapping cognitions related to depressive, anxious, and angry states not specifically linked with the loss participants had confronted), summed scores on the GCQ-C (tapping loss related negative thoughts) would be more strongly associated with the degree to which children and adolescents experienced impairments in functioning as a result of the loss. Finally, we explored the extent to which scores on both questionnaires varied as a function of demographic and loss related variables (e.g., time since loss and cause of death).

## Method

### Participants and Procedure

Participants were recruited from two sources, as described below. Children, adolescents, and their parents from both samples completed the GCQ-C and additional questionnaires. All participants had the Dutch nationality. No data were collected on socio-economic status. Table 1 shows the characteristics of all participants. In total, 151 children aged 8–18 years, completed the measures.

**Table 1:** Demographic characteristics, loss-related characteristics, and scores on prolonged grief and depression measures across samples

	Sample 1	Sample 2	Total Sample
	*N* = 83	*N* = 68	*N* = 151
*Demographic characteristics*			
Gender [*N* (%)]			
Male	36 (43.4)	30 (44.1)	66 (43.7)
Female	47 (56.6)	38 (55.9)	85 (56.3)
Age (*M* (SD))	11.08 (2.54)	13.24 (2.75)	12.05 (2.84)
*Loss*-*related characteristics*			
Deceased is [*N* (%)]			
Parent	74 (89.2)	55 (80.9)	129 (85.4)
Sibling	7 (8.4)	4 (5.9)	11 (7.3)
Other relative	2 (2.4)	9 (13.3)	11 (7.3)
Cause of death is [*N* (%)]			
Illness	31 (37.3)	40 (58.8)	71 (47.0)
Traumatic (accident, suicide, homicide)	17 (20.5)	17 (25.0)	34 (22.5)
Sudden medical cause (e.g. heart attack)	35 (42.2)	11 (16.2)	46 (30.5)
Death was expected by participants?			
Yes	19 (22.9)	26 (38.2)	45 (30.2)
No	62 (74.7)	42 (61.8)	104 (69.8)
Time since loss in months [*M* (SD)]	30.84 (20.40)	37.94 (39.85)	34.01 (30.74)
*Symptom scores*			
IPG-C	52.24 (12.14)	52.69 (11.99)	52.44 (12.03)
CDI	10.50 (7.71)	12.39 (6.82)	11.35 (7.36)

There were occasional missing values for some variables

*CDI* Children’s Depression Inventory, *IPG-C* Inventory of Prolonged Grief for Children

#### Sample 1

Sample 1 was recruited through collaboration with a national grief support group for children in the Netherlands that offers counseling and advice to parents and children about grief and bereavement and that organizes support-weekends for children and their parent(s). During the period of data-collection for the present study, all families that had a child or adolescent applying for such a weekend were sent a letter. The letter included a description of the study and a stamped refusal card. If no refusal card was received in 2 weeks, the family was contacted and—if parent(s) and child agreed to participate—a home visit was planned. In total, 121 families were approached and parents and children of 83 (68.6 %) agreed to participate. Trained graduate students conducted home visits. During the visits, aims of the study were explained and questionnaires were administered. Before completion of the questionnaires, assent was obtained from children (aged 8–12 years), whereas informed consent was obtained from parents and adolescents (aged 13–18 years). If needed, the students could help with completion of the questionnaires (e.g., read questions for dyslectic youth). Sample 1 included 83 children. Thirty children and adolescents were on a waiting list for a support group. They filled in the GCQ-C a second time, approximately 6 weeks later (*M* = 5.67, SD = 1.67 weeks, range 3–9 weeks) and a third time after 30 weeks (*M* = 28.97, SD = 2.12 weeks, range 25–34 weeks). These children and adolescents did not receive psychotherapy or other professional aid during this time interval.

#### Sample 2

The second sample was recruited via an outpatient clinic of Utrecht University. During the period of data-collection for the present study, consecutive patients aged 8–18 years who reported loss-related emotional problems among the complaints they sought help for, were invited to participate. In total, 68 children were approached and they all agreed to participate. Participants completed questionnaires, accompanied by their therapist. Before entering the study, assent was obtained from children, informed consent from parents and adolescents. Sample 2 included 68 children and adolescents; they were not asked to complete measures a second time.

### Measures

All participants completed the GCQ-C, together with a brief questionnaire about demographic and loss-related (e.g., cause of death) variables. Participants also rated their coping self-efficacy. In addition, children completed the Inventory of prolonged grief for children (IPG-C), Child PTSD Symptom Scale (CPSS), Children’s Depression Inventory (CDI) and Children’s Automatic Thoughts Scale (CATS). Children aged 11–18 years from both samples also completed the Youth Self Report (YSR). In both samples, at least one of the parents completed the Child Behavior Checklist/6–18 (CBCL).

#### Grief Cognitions Questionnaire for Children (GCQ-C)

The GCQ-C is based on the 38-item GCQ for adults developed by Boelen et al. (2003; see also Boelen and Lensvelt-Mulders 2005). A confirmatory factor analysis revealed that this adult GCQ included nine categories of negative bereavement related thoughts: global negative beliefs about (1) the self, (2) the world, (3) life, and (4) the future; negative cognitions about (5) self-blame; (6) other people’s responses after the loss, and (7) the appropriateness of one’s grief reactions; and (8) cognitions about the importance of cherishing the pain of the loss and (9) threatening interpretations of one’s reactions to the loss (Boelen and Lensvelt-Mulders 2005). We constructed the GCQ-C, the adaptation for children, in three steps.

In the first step, two experts in clinical child psychology were asked to simplify the wording of the 38 items of the adult GCQ, and to add items tapping the nine categories of cognitions distinguished in the adult GCQ, if so needed. This resulted in provisional list of 55 items. In the second step, we evaluated this draft version with two independent reviewers (both clinical child psychologists with many years of working experience with bereaved children) and 20 children who were not included in further analyses of this study with a mean age of 11.04 (SD = 2.05) years. We interviewed them to obtain their opinion about the wording of items (i.e., Did they understand what we asked them?). Comments about the comprehensibility of questionnaires led to minor changes in wording. Importantly, the experts suggested to include a number of items tapping ‘death anxiety’ (e.g., “I’m afraid that my (foster)parent or another loved one also will die very soon”, “I always think about the possibility that I also can die very soon”). This theme was not included in the adult GCQ but was considered to be relevant to the psychological experiences of bereaved children. It was our intention to develop a short questionnaire with two or three items for all categories of cognitions distinguished in the adult GCQ. Therefore, in the third step of this process we asked the two expert reviewers and 20 children and adolescents to select 1–3 items that were considered to have best face validity, for each of the 10 categories of cognitions that were tapped by the list. This resulted in the final 20-item version of the GCQ-C that is included in the Appendix. Respondents were instructed to rate the frequency of each thought in the preceding 2 weeks on a 3-point Likert scale with categories 0 = *hardly ever*, 1 = *sometimes*, and 2 = *always*. Specifically, instructions were as follows: “Below are possible thoughts that bereaved children can have. Indicate which answer fits best to your experience in the past month. You can answer as follows: 0 = hardly ever, 1 = sometimes and 2 = always.”

#### Inventory of Prolonged Grief for Children (IPG-C)^1^

The IPG-C is a 30-item measure to assess PGD symptoms in children. Respondents rate the frequency of each symptom in the preceding month on 3-point scales (1 = *hardly ever*, 2 = *sometimes*, 3 = *always*). The IPG-C also includes one item (item 28) assessing impairment in functioning as a result of the loss [i.e., “I am doing worse (in school and with friends) since s/he died”]. A study by Spuij et al. (2012a) showed psychometric properties of this questionnaire; the measure has good internal consistency, stability, and concurrent validity. In this study Cronbach’s alphas were 0.93 (Sample 1) and 0.91 (Sample 2), respectively.

#### Child PTSD Symptom Scale (CPSS)

Symptoms of PTSD were assessed with the CPSS, a 24-item self-report questionnaire. It was originally constructed by Foa et al. (2001; Dutch translation Engelhard 2005) and further examined by, e.g., Nixon et al. (2013). The first 17 items correspond to PTSD symptoms as defined in the Diagnostic and Statistical Manual of mental disorders (DSM-IV-TR; APA 2000). Respondents rated the occurrence of these symptoms on 4-point scales ranging from 0 = *not at all/only once a week* to 3 = *almost always/five or more times a week*. The index-event was defined as “the death of your loved one”. The CPSS also includes 7 items assessing functional impairment that is experienced as a result of these symptoms, rated as absent or present. Psychometric properties of the CPSS are adequate (Foa et al. 2001). The internal consistencies (Cronbach’s alpha) for the 17 items tapping symptoms were 0.94 in Sample 1 and 0.90 in Sample 2, respectively. The alphas for the 7 items tapping functional impairment were 0.69 in Sample 1 and 0.53 in Sample 2, respectively.

#### Children’s Depression Inventory (CDI)

The CDI developed by Kovacs (2003) measures symptoms of depression. It contains 27 groups of three statements representing depressive symptoms at increasing levels of severity, scored from 0 = *symptom absent* to 2 = *symptom present always/most of the time*. For all 27 items, respondents select the statement that best describes the severity of the symptom during the preceding week. Items are summed to form an overall depression severity score. The original English (cf. Cole and Martin 2005) and Dutch versions of the CDI (Timbremont et al. 2008) have adequate psychometric properties. In the present samples, the Cronbach’s alpha in Sample 1 was 0.87 and 0.83 in Sample 2.

#### Children’s Automatic Thoughts Scale (CATS)

The CATS (Schniering and Rapee 2002) assesses automatic thoughts in youth associated with a broad spectrum of negative emotional states. It consists of 40 items in four subscales: (1) Physical Threat (e.g., “There is something very wrong with me”), (2) Social Threat (e.g., “I look like an idiot”), (3) Personal Failure (e.g., “I’m worthless”) and (4) Hostile Intent (e.g., “Most people are against me”). The CATS is tested in multiple studies with clinical and non-clinical populations (Micco and Ehrenreich 2009; Schniering and Lyneham 2007; Schniering and Rapee 2002, 2004). It is a reliable and valid measure that shows good convergent validity with related anxiety and depression scales. The CATS yielded Cronbachs’s alphas of 0.91 (Physical Threat), 0.93 (Social Threat), 0.94 (Personal Failure) and 0.89 (Hostile Intent) in Sample 1, and 0.91 (Physical Threat), 0.87 (Social Threat), 0.90 (Personal Failure) and 0.85 (Hostile Intent) in Sample 2.

#### Coping self-efficacy

We formulated one item to assess coping self-efficacy: “Please rate how well you feel that you have processed this loss”, rated on a scale ranging from 0 = *I have not processed the loss at all* to 10 = *I have processed the loss very well*.

#### Child Behavior Checklist/6–18 (CBCL)

The CBCL is a measure of emotional and behavioral problems of children and adolescents ages 6–18 years constructed by Achenbach and Rescorla (2001; Dutch translation, Verhulst et al. 1996). It includes 118 items, representing different problem areas (e.g., anxious, depressive, somatic symptoms, aggressive behavior, and attentional problems). The measure is completed by parents. Items are rated on 3-point scales (0 = *not true*, 1 = *somewhat or sometimes true*, 2 = *very true/often true*) and represent eight different problem areas. Scores on some of these areas can be summed to obtain indices of Internalizing Problems and Externalizing Problems, whereas the summed score of all items represents a Total Problem score. Psychometric properties of the original (Achenbach and Rescorla 2001) and Dutch versions (Verhulst et al. 1996) are adequate. At least one of the parents of each participant completed the CBCL. In case both parents completed the scale, we only used data from one randomly selected parent. This random selection was justified, given that correlations between the father’s and mother’s version, if available, were high.^2^ The internal consistencies of the Internalizing subscale, the Externalising subscale, and the Total scale in the combined children samples from Sample 1 were 0.83, 0.93, and 0.94 respectively, and in Sample 2 these were 0.85, 0.87, and 0.93 respectively.

#### Youth Self Report (YSR)

The YSR, developed by Achenbach and Rescorla (2001), is a 120 item measure of emotional and behavioral problems among youngsters between 11 and 18 years. Its items are comparable to those of the CBCL except that they are written in the first person and completed by youngsters between 11 and 18 years of age. Items representing problems are rated using a forced-choice response format (0 = *not true*, 1 = *somewhat/sometimes true*, 2 = *very/often true*). As with the CBCL, indices of internalizing problems and externalizing problems, as well as a total problem score can be obtained from the measure. Psychometric properties of the original version (Achenbach and Rescorla 2001) and the Dutch version (Verhulst et al. 1997) are adequate. In the present study the YSR was only completed by adolescents (participants aged 13–18 years) from both samples. The YSR yielded Cronbach’s alpha of 0.88 (Internalizing Problems), 0.83 (Externalizing Problems), and 0.90 (Total scale) in Sample 1 and 0.85 (Internalizing Problems), 0.84 (Externalizing Problems) and 0.91 (Total scale) in Sample 2.

### Statistical Analyses

First, we examined the dimensionality of the GCQ-C, using exploratory factor analysis with a varimax rotation, implemented in Mplus 4 with maximum likelihood estimation (Muthén and Muthén 2007). In order to have a sufficient number of participants for this analyses, data of both samples (*N* = 151) were combined. Secondly, the internal consistency and temporal stability of the GCQ-C were examined. Next, several predictions were tested with respect to the validity of the GCQ-C. With respect to the *concurrent validity*, it was expected that negative cognitions as tapped by the GCQ-C would be significantly (positively) correlated with the severity of prolonged grief (IPG-C), depression severity (CDI), PTSD severity (CPSS), and internalizing and externalizing problems as measured by the CBCL and YSR, and significantly inversely correlated with coping self-efficacy (cf. Haine et al. 2008; Melhem et al. 2007; Spuij et al. 2012a).

With respect to the *convergent and divergent validity*, it was expected that the GCQ-C would be more strongly associated with the CATS scales “Physical Threat”, “Social Threat”, and “Personal Failure” than with the CATS scale “Hostile Intent” (see Schniering and Lyneham 2007). We also expected GCQ-C scores to be more strongly associated with the IPG-C item that assesses impairment in functioning as a result of the loss and the summed CPSS items tapping functional impairment, than summed scores on the CATS. Finally, we used correlational analyses and analysis of variance to explore the extent to which scores on the GCQ-C differed as a function of demographic and loss related variables that were assessed (see Table 1).

## Results

### Characteristics of the Study Samples

Table 1 shows background and loss related characteristics of both samples. As can be seen, most participants had lost a parent. Most losses were due to illness and most losses were experienced as being unexpected. Scores on the IPG-C (minimum 30, maximum 90) and the CDI are also shown in Table 1. Scores on the IPG-C did not differ between Sample 1 (*M* = 52.24, SD = 12.14) and Sample 2 (*M* = 52.69, SD = 11.99); *t*(149) = −0.228, *p* = 0.74). In comparison with reference groups from Timbremont et al. (2008) scores on the CDI in both samples fell in the subclinical range.

### Dimensionality

The exploratory factor analysis resulted in the emergence of four factors with eigenvalues greater than 1.00 (i.e., 11.76, 1.45, 1.19, 1.08). However, there were reasons to conclude that the GCQ-C items are best characterized as one factor. Firstly, the first factor explained almost 45 % of the variance in the GCQ-C with the second through fourth factor each adding very little the variance explained by the first factor. Secondly, inherent to that, the scree plot revealed a clear break after the first component. Thirdly, as shown in Table 2, in the one factor solution, all 20 items had factor-loadings ≥0.50. Finally, in the models with more than one factor, several items loaded highly on more than one factor and factors could not be interpreted in a meaningful way. Overall, the findings suggested that, within the present dataset, the GCQ-C items clustered together into one underlying dimension of “overall loss related negative thinking”.

**Table 2:** Abbreviated items of the GCQ-C and factor-loadings in the one-factor solutions

		Factor-loadings in sample 1 and 2 (*N* = 151)
1	Since s/he died, I think of myself as a weak person	0.88
2	Since s/he is dead, I am of no use to anyone anymore	0.87
3	Since s/he is dead, I think the world is bad	0.61
4	Since s/he is dead, I think the world is worthless	0.86
5	I think that others should pay attention to how I am doing	0.50
6	I don’t show others what I think and feel, because I am afraid that that only makes things worse for them	0.68
7	I should have seen to it that s/he would not have died	0.75
8	I blame myself for not having cared for him/her better than I did	0.82
9	I don’t think that I will ever feel better; I don’t feel confidence about the future without him/her	0.88
10	I think that the future will be no fun without him/her	0.82
11	As long as I am sad, I don’t have to let him/her go	0.66
12	I want to hold on to my sorrow for as long as possible	0.60
13	It is not nice toward him/her, when I will begin to feel less sad	0.72
14	I think that my feelings about this loss are not normal	0.75
15	My life has little to offer now s/he died	0.85
16	My life is worthless since s/he died	0.82
17	Sometimes I think that something is wrong with me, because I feel so sad about his/her death	0.83
18	When I think about his death, I feel frightened about all the things I feel	0.87
19	I continue to think that the thing that happened to him/her can also happen to me	0.79
20	Ever since s/he died, I continue to think that I can also die	0.78

### Internal Consistency

Cronbach’s alpha for the 20 items of the GCQ-C was 0.95 in Sample 1 (*n* = 83), 0.89 in Sample 2 (*n* = 63), and 0.93 in the combined sample (*N* = 151). In line with the results of the exploratory factor analysis, in the combined sample the item-total correlations were all positive and ranged from 0.42 (item 5: “I think that others should pay attention to how I am doing.”) to 0.87 (item 18: “When I think about his death, I feel frightened about all the things I feel.”). In the combined sample, the internal consistency did not increase with a deletion of a single item.

### Temporal Stability

Thirty children completed the GCQ-C 3–9 weeks after the first time and 25–34 weeks after the first assessment. The test–retest correlation for the 30 children between scores at assessment 1 and assessment 2 was *r* = 0.84 (*p* < 0.001). The test–retest correlation for the scores at assessment 1 and assessment 3 was *r* = 0.73 (*p* < 0.001).

### Concurrent Validity

Correlations of the GCQ-C with the IPG-C, CDI, CPSS, and CBCL scores are shown in Table 3. As predicted, the GCQ-C total score was significantly and positively correlated with indices of PGD (IPG-C), depression (CDI), posttraumatic stress (CPSS) and Internalizing Problems (CBCL-Internalizing); correlations were all large. Unexpectedly, GCQ-C scores were not significantly correlated with the CBCL-Externalizing scale and the CBCL Total Problems score. Also as we expected, higher scores on the GCQ-C were correlated with lower scores on the item tapping coping self-efficacy; *r* = −0.24, *n* = 147, *p* < 0.01.

**Table 3:** Correlations between study measures in children

	GCQ-C
	*r*
Prolonged grief disorder (IPG-C)	0.76**
Depression (CDI)	0.67**
Posttraumatic stress (CPSS)	0.78**
CATS-physical threat	0.78**
CATS-social threat	0.66**
CATS-personal failure	0.78**
CATS-hostile intent	0.54**
Internalising problems (CBCL)	0.18*
Externalising problems (CBCL)	0.02
Total problems (CBCL)	0.14
Internalising problems (YSR)	0.74**
Externalising problems (YSR)	0.30**
Total problems (YSR)	0.63**

Samples sizes differ due to occasional missing values; IPG-C, CDI, CPSS and CATS were filled in by *N* = 151, CBCL by 144 parents and YSR by 92 adolescents

*CDI* Children’s Depression Inventory, *CBCL* Child Behaviour Checklist, *CPSS* Child Posttraumatic Stress Disorder Symptom Scale, *IPG-C* Inventory of Prolonged Grief for Children, *YSR* Youth Self Report

* *p* < 0.01; ** *p* < 0.001

### Convergent and Divergent Validity

Correlations of the GCQ-C with the CATS subscales (*N* = 151) are also shown in Table 3. As predicted, the GCQ-C was more strongly associated with Personal Failure (*r* = 0.78), Physical Threat (*r* = 0.78) and Social Threat (r = 0.66), than with the Hostility subscale (*r* = 0.54, all *p*s < 0.01). Differences between correlations were significant (*r* = 0.78 vs. *r* = 0.54, *z* = −4.97 *p* < 0.001; *r* = 0.78 vs. *r* = 0.54, *z* = −4.87 *p* < 0.001; *r* = 0.66 vs. *r* = 0.54, *z* = −2.11, *p* < 0.02.

Finally, both the GCQ-C total score and the summed score on the CATS were significantly associated with the IPG-C item that assesses impairment in functioning as a result of the loss. However, in contrast with what was expected, the correlation of the GCQ-C total score with functional impairment (*r* = 0.46, *p* < 0.001) was equal to the correlation of the CATS score with functional impairment (*r* = 0.45, *p* < 0.001). Consistent with that, both the correlation of the GCQ-C total score with the summed functional impairment items of the CPSS and the correlation of the CATS score with this index were *r* = 0.53, *p* < 0.001.

### Demographic and Loss-Related Correlates of GCQ-C Total Scores

Scores on the GCQ-C did not vary as a function of time from loss, age, gender, relationship to the deceased, cause of death, and whether or not the death was expected (all *p*s > 0.08).

## Discussion

The aims of the present study were to examine psychometric properties of the Grief Cognition Questionnaire for Children (GCQ-C) and, more generally, to enhance knowledge about maladaptive thinking in bereaved children (aged 8–18 years). The GCQ-C was modeled after the adult Grief Cognitions Questionnaire (Boelen et al. 2003) and designed to measure bereavement-related negative cognitions that are assumed to play a role in the development and persistence of childhood PGD and other symptoms of post loss psychopathology.

With regard to the dimensionality of the GCQ-C, outcomes of an exploratory factor analysis showed that the GCQ-C represented one underlying dimension of negative loss-related cognitions. This finding is inconsistent with research based on the adult GCQ, in which negative cognitions clustered into different factors representing different themes, including negative cognitions about the self, life, the future, and catastrophic misinterpretations of grief reactions (Boelen and Lensvelt-Mulders 2005). These findings suggest that there are differences between thinking patterns of bereaved children and adults such that bereaved children differentiate between different types of cognitions associated with loss less than adults do. Indeed, it is possible that adults distinguish between different themes (including the view of themselves, their lives, and futures) more clearly, such that some of these themes are appraised more negatively than others, whereas children’s negative appraisals generalize across different themes. In a related vein, it is possible that children do not necessarily experience less sadness or grief following loss compared to adults, but are less aware of the precise content of their cognitions that underly these feelings. It is also possible that self-report questionnaire are adequate means to assess different types of cognitions in adults, but may be less suitable to assess nuanced differences in cognitions about different themes in children. More research on this topic in larger populations is needed to gain more knowledge about the phenomenology of maladaptive thinking in bereaved children and differences with maladaptive thoughts patterns in adults. Reliability analyses showed that the GCQ-C had adequate internal consistency and temporal stability.

With respect to the concurrent validity, the GCQ-C total score was significantly and positively correlated (moderate to strong correlations) with self-report indices of PGD, depression and PTSD. It was also significantly, but with a weak correlation, correlated with internalizing problems as reported by parents, but not with externalizing problems. This finding is in line with prior research (e.g. Melhem et al. 2007; Spuij et al. 2012a) indicating that clinicians should ask children as informants about their grief related cognitions. Furthermore, as expected, higher scores on the GCQ-C were correlated with lower scores on the perceived ability to cope with the loss indicating that more negative cognitions associated with the loss coincided with a more negative view of children about their own ability to deal with the challenges brought about by the loss.

Several findings supported the convergent and divergent validity of the GCQ-C. GCQ-C scores were significantly related to automatic thoughts associated with a broad spectrum of negative emotional states, tapped by the CATS (Schniering and Rapee 2002); as predicted, the GCQ-C was more strongly associated with CATS subscales measuring cognitions associated with personal failure, physical threat, and social threat, than with the CATS subscale measuring cognitions associated with Hostility. There were no differences between the GCQ-C and the CATS in terms of their associations with indices of functional impairment associated with the loss.

It is noteworthy that endorsement of negative cognitions tapped by the GCQ-Q did not differ as a function of the background and loss related variables that we assessed. These findings accord with prior findings of a weak linkage between background and loss-related variables and PGD symptoms (Spuij et al. 2012a) and suggest that background and loss-related variables generally are not associated with high levels of loss related psychological problems.

Some limitations should be taken into account when interpreting the present findings. First, the sample size (*N* = 151) may be considered somewhat small for exploratory factor analyses. Notably though, Guadagnoli and Velicer (1988) argued that a factor is reliable, regardless of sample size, when it has four or more loadings greater than 0.6 and that with all communalities above 0.6 samples less than 100 may be adequate and with communalities in the 0.5 range samples between 100 and 200 can be good enough. This is why we decided that although the *N* was quite small, it was worth to explore the factors in this sample. Second, given the variety of methods of recruitment, most of the analyses that we conducted relied on a rather homogeneous group, as most children lost a parent who died from cancer. Maybe there are differences in origin and severity of grief cognitions between subgroups (e.g., children who suffered from traumatic loss, children who lost a sibling or other relative) that were unidentified in the current study because of sample size constraints. Third, CBCL’s for participants were completed by parents. Because of their own grief, these parents possibly were not the most reliable informants of their children’s problems. Thus, it would be relevant for future studies to correlate grief cognitions of children and PGD severity also with indices of children’s problems obtained from other, more distant informants such as teachers. Fourth, the present study tested a Dutch version of the GCQ-C. Although it is conceivable that the present findings are generalizable to other Western—including English-speaking—cultures, the psychometric properties of versions of the questionnaire in other languages remain to be tested. As a related point, the cultural background of participants had a Western origin. Thus, generalization of the present findings to non-Western subgroups or religions remains to be determined. A final limitation of this study was its cross-sectional design that precluded any conclusions about the direction of causality between negative cognitions and emotional problems. It would be interesting for future research to use prospective-longitudinal designs to disentangle the linkage between negative thinking and emotional problems further.

Notwithstanding these considerations, results of the present study indicate that the GCQ-C is a promising measure for the assessment of negative loss-related cognitions in children and adolescents confronted with bereavement. As such it may be used in future studies on the role of cognitive variables in emotional problems after bereavement. To our knowledge, this is the first study that provides evidence for a strong link between negative thinking associated with different themes on the one hand, and elevated PGD and other symptoms of depression and PTSD on the other hand. Moreover, the measure may be useful in clinical practice for the identification of cognitions that are important targets of the treatment of PGD-symptoms. Given the lack of effective interventions for childhood grief (Currier et al. 2007), it is important to know which factors can mediate emotional problems in children after a loss. We recently conducted two studies that provided preliminary evidence that cognitive behavioral interventions are fruitful in ameliorating PGD symptoms and other symptoms of distress in bereaved children and adolescents (Spuij et al. 2013, 2015). The findings of these prior studies and the current study suggest that negative thinking plays an important role in childhood PGD and should be targeted in the treatment of this condition. This is in line with research from Meiser-Stedman et al. (2009) who showed that maladaptive cognitions in children with PTSD are causally implicated in the unfolding and maintenance of posttraumatic stress responses over time and that these should be the focus of treatment of PTSD in children.

## Appendix

### English translations of the Grief Cognitions Questionnaire for Children (GCQ-C)

Below are possible thoughts that bereaved children can have. Indicate which answer fits best to your experience in the past month.

You can answer as follows: 0 = hardly ever, 1 = *sometimes* and 2 = *always.*

**Table:**

Global negative thoughts about the self	1	Since s/he died, I think of myself as a weak person
	2	Since s/he is dead, I am of no use to anyone anymore
Global negative thoughts about the world	3	Since s/he is dead, I think the world is bad
	4	Since s/he is dead, I think the world is worthless
Negative thoughts about the other people’s responses after the loss	5	I think that others should pay attention to how I am doing
	6	I don’t show others what I think and feel, because I am afraid that that only makes things worse for them
Negative cognitions about self-blame	7	I should have seen to it that s/he would not have died
	8	I blame myself for not having cared for him/her better than I did
Global negative thoughts about the future	9	I don’t think that I will ever feel better; I don’t feel confidence about the future without him/her
	10	I think that the future will be no fun without him/her
Cognitions about cherishing the pain	11	As long as I am sad, I don’t have to let him/her go
	12	I want to hold on to my sorrow for as long as possible
	13	It is not nice toward him/her, when I will begin to feel less sad
Appropriateness of one’s grief reactions	14	I think that my feelings about this loss are not normal
Global negative thoughts about the world	15	My life has little to offer now s/he died
	16	My life is worthless since s/he died
Threatening interpretations of one’s reactions to the loss	17	Sometimes I think that something is wrong with me, because I feel so sad about his/her death
	18	When I think about his death, I feel frightened about all the things I feel
Death anxiety	19	I continue to think that the thing that happened to him/her can also happen to me
	20	Ever since s/he died, I continue to think that I can also die

## Footnotes

1 Spuij et al. (2012) developed the IPG-C and IPG-A for children and adolescents, respectively. Both inventories had 30 items and minor differences in wording in 17-items. In the current study, we only used the IPG-C—both for children and adolescents. Before data-collection for the present study started, we asked 20 children and adolescent for their opinion about the GCQ-C items [as reported in the section “Grief Cognitions Questionnaire for Children (GCQ-C)”]. The adolescents in this group were asked whether they considered the items of the IPG-C understandable. They all confirmed the usefulness of the IPG-C items for their age group.

2 Specifically, for 21 children from Sample 1 and 2 data were available from both parents. The correlations between scores of the fathers and mothers for the Internalising score was *r* = 0.60 *(p* < 0.01), for the Externalising score was *r* = 0.73 (*p* < 0.001), and for the Total score was *r* = 0.61 (*p* < 0.01).
